# Prevalence of Acute Q Fever Among High-Risk Patients With Fever and Pneumonia Symptoms in Western Iran

**DOI:** 10.1155/jotm/6685577

**Published:** 2025-08-11

**Authors:** Amjad Ahmadi, Rasool Nasiri Kalmarzi, Behzad Mohsenpour, Parisa Esmaeili, Mina Latifian, Ahmad Ghasemi, Rashid Ramazanzadeh, Himen Salimizand, Daem Roshani, Saber Esameili, Ehsan Mostafavi

**Affiliations:** ^1^Zoonoses Research Center, Research Institute for Health Development, Kurdistan University of Medical Sciences, Sanandaj, Iran; ^2^Health Metrics and Evaluation Research Center, Research Institute for Health Development, Kurdistan University of Medical Sciences, Sanandaj, Iran; ^3^Lung Diseases and Allergy Research Center, Research Institute for Health Development, Kurdistan University of Medical Sciences, Sanandaj, Iran; ^4^WHO Collaborating Center for Vector-Borne Disease, Department of Epidemiology and Biostatics, Research Centre for Emerging and Reemerging Infectious Diseases, Pasteur Institute of Iran, Tehran, Iran; ^5^National Reference Laboratory for Plague, Tularemia and Q Fever, Research Centre for Emerging and Reemerging Infectious Diseases, Pasteur Institute of Iran, Akanlu, Kabudarahang, Hamadan, Iran; ^6^Reference Health Laboratories, Ministry of Health and Medical Education, Tehran, Iran; ^7^Department of Microbiology, Faculty of Medicine, Ardabil University of Medical Sciences, Ardabil, Iran; ^8^Department of Vaccinology and Immunotherapeutics, School of Public Health, University of Saskatchewan, Saskatoon, Saskatchewan, Canada

**Keywords:** acute Q fever, *Coxiella burnetii*, Iran, pneumonia, Q fever

## Abstract

**Background:** Q fever is a zoonotic bacterial infection with worldwide distribution. Based on seroepidemiology studies among the human population and also serological and molecular surveys of animals, Q fever is an endemic disease in Iran. However, the status of acute Q fever in many parts of Iran is still unknown. This study aimed to investigate acute Q fever among high-risk patients with fever and pneumonia symptoms in Western Iran.

**Methods:** In this survey, 96 patients were included in the study in Kurdistan Province who had symptoms of suspected pneumonia or acute Q fever and epidemiological evidence for the risk of Q fever. From each individual, paired acute and convalescent serum samples were taken, and the elevation of the phase II IgG antibody titer against *Coxiella burnetii* was traced by ELISA. Also, molecular detection of *C. burnetii* was done in acute blood samples by real-time PCR.

**Results:** Seven patients (7.3%) were diagnosed with acute Q fever who had seroconversion and a four-fold rise in the phase II IgG antibody titer against *C. burnetii* in their paired sera samples. Also, 22 of 89 (24.7%) individuals with a negative result for acute Q fever had a previous history of exposure to *C. burnetii*. There was a significant relationship between sheep husbandry and a previous history of exposure to *C. burnetii* (*p*=0.04). All molecular tests were negative.

**Conclusion:** The results of this study showed that there are cases of acute Q fever in Western Iran, but it is not considered by the healthcare system or clinicians.

## 1. Introduction

Q fever is a highly infectious zoonotic disease caused by the intracellular bacterium *Coxiella burnetii* [1, 2]. Domestic ruminants such as cattle, goats, and sheep are considered to be the main reservoirs of *C. burnetii*. Infected animals are often asymptomatic, except in very limited cases, leading to miscarriage and infertility in female animals. These animals shed this bacterium into the environment through milk, feces, urine, and birth products. Human infection with *C. burnetii* occurs mainly through the inhalation of aerosols infected with the bacterium. Also, direct contact and consumption of contaminated raw milk and dairy products are the less common routes of transmission of infection to humans [3–5].

The primary infection of *C. burnetii* in humans is asymptomatic in more than 60% of individuals. The symptomatic infections of Q fever in humans are mainly classified into two main forms, including acute Q fever and chronic Q fever. Acute Q fever is mainly a self-limiting and flu-like febrile illness that has nonspecific clinical manifestations including fever, chills, severe headache, cough, weight loss, atypical pneumonia, hepatitis, muscle aches, arthralgia, cardiac involvement, skin rash, and neurological signs [6, 7]. In general, hospitalization and case fatality rates (without proper treatment) of acute Q fever patients are under 5% and 1%-2%, respectively [8]. Persistent *C. burnetii* infection in humans (less than 5% of acute Q fever cases) can lead to a chronic Q fever and may present months to years after an acute infection. Endocarditis is the most important and common clinical manifestation of chronic Q fever that can be life-threatening without proper diagnosis and treatment [9].

The laboratory diagnosis of Q fever is mostly based on serological and molecular methods. The gold standard for laboratory diagnosis includes serological methods such as enzyme-linked immunosorbent assay (ELISA) and indirect immunofluorescence [10, 11]. Recently, ELISA has been used more for the diagnosis of acute Q fever because of its economics and convenience [12].

According to recently available data, Q fever is an endemic disease in Iran, and it is considered an important but neglected disease [13–15]. In recent years, cases of acute Q fever and Q fever endocarditis have been reported in different parts of Iran, illustrating the importance of the disease in the country [15–20]. However, there is still not much information available on the epidemiology of human Q fever clinical cases in some parts of Iran, especially in the western parts. Kurdistan Province is one of the western provinces of Iran that still lacks epidemiological information on human cases of Q fever. In a recent study, 46.6% of domestic ruminants in Kurdistan Province had antibodies against *C. burnetii* [21]. Also, 63% of collected milk samples from dairy animals in this province were positive for *C. burnetii* by the serological method [22]. Molecular analysis using nested PCR and real-time PCR revealed that 34.92% of raw milk samples from dairy animals with a history of abortion in Iran were positive for *C*. *burnetii* [15]. In a seroepidemiological study (2011-2012), the seroprevalence of Q fever was reported at 27.8% in different human populations in Kurdistan Province [21, 23]. On the other hand, a case of Q fever endocarditis has been reported recently in this province [24]. However, data on acute Q fever are not available in Kurdistan Province. Kurdistan Province is one of the livestock centers in Iran, and most of the population of this province is exposed to domestic livestock. Therefore, the prevalence of acute Q fever in this province is not unexpected. The aim of this study was to investigate the prevalence of acute Q fever in high-risk individuals in order to further a better understanding of the epidemiological features of acute Q fever in Kurdistan Province.

## 2. Materials and Methods

### 2.1. Study Area

This study was conducted in Kurdistan Province from 2018 to 2020. This province is 29.137 km^2^ in area, equivalent to 7.1% of the total area of Iran. This province is located on the scattered slopes and plains of the Middle Zagros Mountains. According to the 2016 Population and Housing Census, Kurdistan Province had a population of 1,603,011, of which 66% and 34% were urban and rural residents, respectively.

### 2.2. Sampling

**Figure 1:**
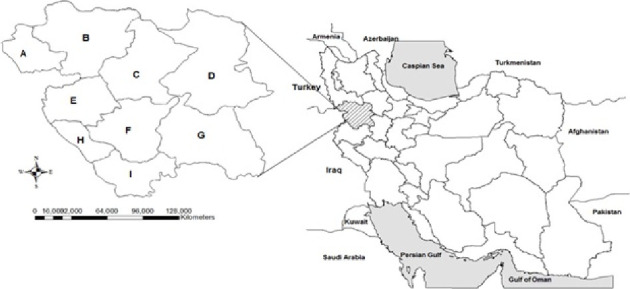
Map of Kurdistan Province and its cities. A: Baneh, B: Saqqez, C: Divandarreh, D: Bijar, E: Marivan, F: Sanandaj, G: Qorveh, H: Sarvabad, and I: Kamyaran.

Samples were collected from patients who visited infectious disease specialists at Be'sat and Tohid Hospitals in the Sanandaj city (Figure 1). This study was performed on 96 patients suspected of having acute Q fever who had the two following criteria:

I. (A) Having a job so that they were in contact with animals or their livestock products (including veterinarians, ranchers, butchers, and farm workers), as well as working in veterinary and medical laboratories, and/or (B) residency in rural areas or close to where livestock are kept.
II. Acute lower respiratory tract infection: fever with at least two other symptoms including chills, headache, atypical pneumonia, dyspnea, or chest pain.

Also, the individuals who participated in this study were people who were willing to refer to the research team for a second blood sample 4 weeks after the initial sampling.

After obtaining written consent from the participants, a questionnaire including demographic characteristics, clinical symptoms, and risk factors was completed for each patient. Then, a 6 mL blood sample was taken from each patient (acute phase sample), and a second blood sample was taken after 4 weeks (convalescent-phase sample) in sterile tubes without anticoagulant. Blood samples were centrifuged for 10 min at 3000 rpm and kept at −20°C after extraction of their sera. Also, about 4 mL of blood from each patient (acute phase sample) was collected in EDTA tubes and kept at 4°C–8°C. All collected samples were transferred to the National Reference Laboratory of Plague, Tularemia, and Q Fever (at the Research Centre for Emerging and Reemerging Infectious Diseases, Pasteur Institute of Iran) for molecular and serological analysis.

### 2.3. Serological Test

Sera samples of acute and convalescent phases of each patient were simultaneously tested for detection of IgG phase II antibodies against *C. burnetii* using a commercial quantitative ELISA kit (Serion ELISA classic, Institut Virion/Serion GmbH, Würzburg, Germany) and according to the manufacturer's instructions. Obtained optical densities (ODs) in the ELISA test were analyzed according to the kit manufacturer's protocol, and levels of IgG phase II antibodies against *C. burnetii* were quantitatively (U/mL) determined. The IgG phase II against *C. burnetii* was considered positive, borderline, and negative when its level was > 30, 20–30, and < 20 U/mL, respectively. If both sera samples of each patient were positive and the four-fold rise was not observed in the IgG phase II levels, it was considered a previous history of exposure to *C. burnetii* (past infection).

### 2.4. Molecular Test

All patients with acute Q fever (serologically confirmed), as well as patients with a history of contact with *C. burnetii* (both sera positive but no increase in antibody levels or seroconversion in paired sera), and 30% of patients whose serological result for acute Q fever is negative were selected for molecular examination of *C. burnetii*. About 200 μL of each selected blood sample was used for DNA extraction using the High Pure PCR Template Preparation Kit (Roche, Germany) according to the manufacturer's instructions. The extracted genomic DNAs were stored at −20°C until use.

The extracted DNA samples were evaluated by real-time PCR targeting the IS1111 transposase elements in the genome of *C. burnetii* (Table 1). Real-time PCR reactions were 20 μL and included 10 μL of 2x RealQ Plus Master Mix for Probe (AmpliQon, Denmark), 900 nM of each primer, 200 nM probe, 4 μL of extracted DNA template, and double-distilled water [25]. Real-time PCR was performed on the Corbett 6000 Rotor-Gene system (Corbett, Victoria, Australia). Double-distilled water and DNA of the Nine Mile strain (RSA 493) were used as negative and positive controls in real-time PCR, respectively. The real-time PCR amplification program was 10 min at 95°C, followed by 45 cycles of 15 s at 94°C and 60 s at 60°C. The results of the real-time PCR test were generated using Rotor-Gene Q 2.3.5 software (QIAGEN).

### 2.5. Data Analysis

The collected data were analyzed using SPSS software Version 16 (SPSS Inc., Chicago, IL, USA). The chi-square test was used to compare epidemiological variables. All results were considered statistically significant if the *p* value was equal to or less than 0.05 and marginally significant (very weak correlation) if the *p* value was between 0.05 and 0.1.

### 2.6. Ethical Considerations

All protocols and stages of this study were reviewed and accepted by the Medical Ethics Committee of the Kurdistan University of Medical Sciences (IR.MUK.REC.1397.069). Written informed consent was obtained from all the participants in this study.

## 3. Results

Of the 96 patients who participated in the study, 60 (62.5%) were male and 36 (37.5%) were female. The average age of the participants in this study was 45.8 years, and 94.8% of the participants in this study were residents of the village. Fever (100%), pneumonia (100%), cough (65%), headache (55%), fatigue (60%), myalgia (55%), and dyspnea (80%) were the most common clinical symptoms of the participants in this study.

Using the laboratory criteria in this study, acute Q fever was diagnosed in 7 of 96 patients (7.3%). All 7 patients with acute Q fever showed a four-fold increase and seroconversion in IgG phase II antibodies against *C. burnetii* titers. Also, all samples (50 patients) were molecularly negative for *C. burnetii*. The prevalence of acute Q fever was 6 (10%) in men and 1 (2.8%) in women. In addition, the prevalence of acute Q fever was 6.7% in rural residents and 20% in urban residents. A very weak correlation was observed between a history of brucellosis (*p*=0.07) and cattle keeping (*p*=0.07) and acute Q fever. Other demographic characteristics and risk factors were not significantly associated with acute Q fever (Table 2).

Headache (85.7%), fatigue (85.7%), dyspnea (71.4%), and cough (57.1%) were the most common clinical symptoms in 7 patients with acute Q fever in this study. However, none of the clinical symptoms recorded in this study were statistically significantly associated with acute Q fever (Table 3).

In addition, 22 of the 89 (24.7%) participants whose final response to acute Q fever was negative had a history of *C. burnetii*. The seroprevalence of Q fever (previous exposure) was 25.9% in men and 22.8% in women, which was not statistically significant (*p*=0.74). Also, 35.8% of individuals with a history of sheep keeping had previous exposure to *C. burnetii*, which was also statistically significant (*p*=0.03). Three participants (30%) of 10 women with a history of abortion had antibodies to *C. burnetii*, but this risk factor was not statistically significant (*p*=0.52). Other demographic characteristics and risk factors were not significantly associated with previous exposure to Q fever.

## 4. Discussion

This study was conducted to determine the prevalence of acute Q fever in patients with fever and pneumonia suspected of having acute Q fever in Kurdistan Province, and it showed that 7.3% were diagnosed with acute Q fever. The patients with acute Q fever in this study were diagnosed by the serological method (seroconversion and four-fold increase in antibody titer), and the molecular result was negative in all patients. Considering that *C. burnetii* was recently detected in the livestock of this province [21, 22] and also in the seroepidemiological study conducted in this province, it was confirmed that people were exposed to this bacterium [23]. Therefore, this study showed that there are clinical cases of acute Q fever in Kurdistan Province. Therefore, all findings point to the importance of Q fever in Western Iran and Kurdistan Province, and the healthcare system and physicians of this province should consider acute Q fever as a differential diagnosis in patients with pneumonia and fevers of unknown origin for which epidemiologic evidence is available. Q fever should be considered likely. On the other hand, although the mortality rate of acute Q fever is very low (less than 1%), large-scale outbreaks of acute Q fever can lead to public health concerns such as blood transfusions or abortions in pregnant women. In addition, a persistent acute Q fever infection can progress to chronic Q fever (often endocarditis) in up to 5% of cases, and the complications of chronic Q fever are very dangerous if not early diagnosed and treated [26]. Therefore, adequate attention should be paid to the treatment of acute Q fever and the continuous serologic follow-up of acute Q fever cases. This point is also not taken seriously by the healthcare system in Iran, and healthcare workers should be trained and educated accordingly.

Although several molecular and serological studies have been conducted about the detection of *C. burnetii* among animal reservoirs in Iran in recent years, clinical human cases of Q fever are still neglected by physicians and the healthcare system. Most of the identified clinical cases have been in the form of research projects in Iran, and the number of these studies is very limited. Therefore, an accurate assessment of the status of the prevalence of acute Q fever in Iran is not available, and more studies should be done in this field to draw the attention of the health system to the importance of this disease. In the present study, 7.3% of the patients were diagnosed with acute Q fever. In a study in Southeastern Iran (2012), 35.5% of febrile patients were diagnosed with acute Q fever [27]. In another study in Northeastern Iran (2015), 7.6% of patients with fevers of unknown origin were found to be positive for *C. burnetii* infection [16]. Also, in another study in Northern Iran (2016), acute Q fever was diagnosed in 5.4% of the patients studied [28]. In a 2016 study of patients with suspected clinical signs of Q fever in Northwestern Iran, 13.8% of subjects were positive for acute Q fever infection [29]. Comparing the results of this study with the limited results of similar studies conducted in Iran, it appears that acute Q fever has a different prevalence in different regions of Iran. Specific geographical features and climatic conditions may influence the prevalence of acute Q fever in different regions. Therefore, further studies should be conducted in other regions of Iran to fully assess the epidemiological status of this disease in Iran. National and large-scale studies could also be helpful to determine the prevalence of acute Q fever.

Because the clinical signs of acute Q fever are nonspecific, it is not possible to make a definitive diagnosis based on clinical signs alone. In this study, fever, headache, fatigue, dyspnea, and cough were the most common clinical signs in 7 patients with acute Q fever, but none of the observed clinical signs was statistically significant. The results of other similar studies in Iran were consistent with our findings [28, 29]. On the other hand, although there was a very weak correlation between the history of brucellosis and cattle kept with acute Q fever, not all risk factors examined in this study were significant. One of the reasons why no significant risk factor for acute Q fever was found in our study may have been the small sample size and the small number of positively identified cases. Another possible reason is that in the present study, 94.7% of the participants lived in rural areas and somehow faced all kinds of risk factors, which made it difficult to find an effective risk factor for acute Q fever. If the control group is also considered in this study (such as blood donors), it may be easier to identify potential risk factors. It is suggested that future studies consider the aforementioned limitations when searching for risk factors.

In the present study, 24.7% of the individuals had *C. burnetii* phase II IgG antibodies, which were considered previous exposure (previous infection) to *C. burnetii*. The prevalence of *C. burnetii* phase II IgG antibodies in the present study is much higher than the prevalence in the 2011-2012 study conducted in Kurdistan Province (14.5%) [23]. The reason for the increased prevalence of seroepidemiology compared to the above study may be that *C. burnetii* has become more widespread in livestock over time in Kurdistan Province, increasing human contact with this bacterium. Husbandry and contact with sheep in this study were significant risk factors for previous exposure to *C. burnetii*. Also, the previous exposure to *C. burnetii* in this study is slightly lower than that in similar studies in other provinces, so the previous exposure in Tabriz (Northwestern Iran) was 32% [29] and that in Ilam (Western Iran) was 26.4% [30]. The nature of the geographical area as well as the different prevalence of *C. burnetii* infection in different regions could be the reason for this difference in seroprevalence.

The overall seroprevalence of acute Q fever in Iran is estimated at 20.8%, indicating widespread exposure to *C. burnetii* [31]. There are significant regional differences in seroprevalence of Q fever among high-risk populations in Iran: southeastern (68%), northwestern (13.8%), western (27.83%), and eastern (25.55%) [29, 31].

In alignment with Iran's neighboring countries, Q fever has been documented in Afghanistan (34.4%), Iraq (22.2%) [31], and Northern Turkey (12.3%–32%) [32]. Several other countries show varying prevalence rates. Different rates of Q fever seroprevalence were reported from other countries, such as 25.6–53.3% in Egypt, 44.4% in Jordan, 20.2% in Lebanon, 16% in Saudi Arabia, 9.8% in Oman, and 8.5% in Tunesia [31]. The mortality rate among hospitalized patients with acute Q fever in Netherlands is approximately 1% [29, 33]. Comparable research in other countries has reported varying molecular prevalence rates of *C. burnetii* among acute febrile patients: 0.4% in Senegal [34], 4.5% in India [35], and 14.1% in Poland [36]. In the United States, seroprevalence for *C. burnetii* accounts for approximately 3.1% [37]. The seroprevalence of Q fever in Spain [38], Australia [39], and Denmark [40] was 15.3, 5.2, and 11%, respectively.

In Northwestern Iran, a notable 88.9% of confirmed acute Q fever patients had a history of keeping livestock, reinforcing the strong association between animal contact and disease acquisition. Interestingly, studies found that 77.8% of detected cases were urban residents, contrary to the expectation that rural populations would be more affected [33]. Occupational exposure significantly influences Q fever prevalence across the region. The highest rates appear among herders (62.0%), butchers (31.9%), and farmers (22.5%) [31]. This occupational distribution pattern is consistent with the zoonotic nature of *C. burnetii*, which primarily spreads through contact with infected livestock and inhalation of contaminated aerosols [33]. The prevalence of acute Q fever in Iran shows considerable regional variation but generally falls within midrange compared to neighboring countries. Neighboring countries show a wide spectrum of prevalence rates, from extremely high (Afghanistan and Jordan) to moderate (Iraq and Oman) and relatively lower (Lebanon and Tunisia) [31]. This is higher than the seroprevalence observed in Iran, likely due to differences in occupational exposure and public health measures. In the United States, Q fever is less common but still a concern, particularly in regions with significant livestock farming. A study in California found that 3.1% of the general population had antibodies against *C. burnetii*, with higher rates in individuals with occupational exposure to livestock [41]. This is lower than the seroprevalence observed in Iran, possibly due to differences in livestock density and farming practices.

Findings on Q fever in Iran could significantly influence public health policies and practices by guiding disease surveillance, prevention, and control measures. If studies confirm a high prevalence of Q fever in Iran, authorities may implement stricter regulations on livestock management, promote public awareness campaigns about zoonotic transmission, and encourage safer dairy consumption practices (e.g., boiling or pasteurizing milk). Additionally, identifying high-risk groups such as farmers, butchers, and individuals in close contact with animals could lead to targeted vaccination programs and occupational health guidelines. Strengthening diagnostic capabilities and integrating Q fever into national infectious disease reporting systems would also enhance early detection and outbreak response, ultimately reducing the disease burden in Iran and its neighboring regions.

This study shows that there is an acute Q fever infection in Western Iran. The results of this study, along with evidence from previous studies about *C. burnetii* in Kurdistan Province (including seroprevalence studies among humans and livestock and molecular detection of this bacterium in milk as well as a recent Q fever endocarditis case), indicated that Q fever is an important disease in this province, and some of the epidemiological features of Q fever were identified in Kurdistan Province. Accordingly, the healthcare system in this province must consider Q fever as a health problem. Special training for clinicians in Kurdistan Province should be provided for the diagnosis of Q fever. It is also recommended that, due to the high rate of Q fever seroprevalence in this province, cases of chronic Q fever infection should also be considered by the health system.

## Figures and Tables

**Table 1:** Primers and probe sequences for diagnosis of *C. burnetii* IS1111 gene by real-time PCR.

	Sequence of primers	Product size (base pair)	Reference
Forward-primer	5′-AAAACGGATAAAAAGAGTCTGTGGTT-3′	70 bp	[25]
Reverse-primer	5′-CCACACAAGCGCGATTCAT-3′		
Probe	5′-6-FAM-AAAGCACTCATTGAGCGCCGCG-TAMRA-3′		

**Table 2:** The correlation between the risk factors and demographic traits of individuals with acute Q fever and prior exposure history in Kurdistan Province, 2018–2020.

Variables	Condition	Acute Q fever		*p* value	History of previous exposure to *C. burnetii*		*p* value
		Negative (89)	Positive (7)		Negative (67)	Positive (22)	
Age average		45.87 ± 13.66	45 ± 16.52	0.87	46 ± 15	45.21 ± 9.21	0.81

Gender	Male	54 (60.7%)	6 (85.7%)	0.18	40 (59.7%)	14 (63.6%)	0.74
	Female	35 (39.3%)	1 (14.3%)		27 (40.3%)	8 (36.4%)	

Place of residence	Village	85 (95.5%)	6 (85.7%)	0.26	64 (95.5%)	21 (95.5%)	0.98
	City	4 (4.5%)	1 (14.3%)		3 (4.5%)	1 (4.5%)	

Cattle keeping	Yes	66 (74.2%)	3 (42.9%)	0.07	50 (74.6%)	16 (72.7%)	0.86
	No	23 (25.8%)	4 (57.1%)		17 (25.4)	6 (27.3%)	

Sheep keeping	Yes	53 (59.6%)	6 (85.7%)	0.17	34 (50.7%)	19 (86.4%)	0.03
	No	36 (40.4%)	1 (14.3%)		33 (49.3%)	3 (13.6%)	

Tick biting history	Yes	3 (3.4%)	1 (14.3%)	0.16	3 (4.5%)	0 (0%)	0.31
	No	86 (96.6%)	6 (85.7%)		64 (95.5%)	22 (100%)	

Abortion history	Yes	10 (28.6%)	0 (0%)	0.72	7 (25%)	3 (37.5%)	0.52
	No	25 (71.4%)	1 (100%)		21 (75%)	5 (62.5%)	

Consumption of raw milk	Yes	83 (93.3%)	6 (85.7%)	0.46	62 (92.5%)	21 (95.5%)	0.63
	No	6 (6.7%)	1 (14.3%)		5 (7.5%)	1 (4.5%)	

History of brucellosis	Yes	2 (2.2%)	1 (14.3%)	0.07	1 (1.5%)	1 (4.5%)	0.43
	No	87 (97.8%)	6 (85.7%)		66 (98.5%)	21 (95.5%)	

**Table 3:** Frequency of clinical symptoms in patients with acute Q fever in Kurdistan Province, 2018–2020.

Clinical symptoms		Acute Q fever		*p* value
		Negative (89)	Positive (7)	
Headache	Yes	49 (55.1%)	6 (85.7%)	0.11
	No	40 (44.9%)	1 (14.3%)	

Chills	Yes	22 (24.7%)	1 (14.3%)	0.53
	No	67 (75.3%)	6 (85.7%)	
Chest pain	Yes	43 (48.3%)	3 (42.9%)	0.78
	No	46 (51.7%)	4 (57.9%)	

Cough	Yes	63 (70.8%)	4 (57.1%)	0.44
	No	26 (29.2%)	3 (42.9%)	

Fatigue	Yes	56 (62.9%)	6 (85.7%)	0.22
	No	33 (37.1%)	1 (14.3%)	

Diarrhea	Yes	12 (13.5%)	0 (0%)	0.29
	No	77 (86.5%)	7 (100%)	

Myalgia	Yes	49 (55.1%)	3 (42.9%)	0.53
	No	40 (44.9%)	4 (57.1%)	

Arthralgia	Yes	50 (56.2%)	3 (42.9%)	0.49
	No	39 (43.8%)	4 (57.15)	

Dyspnea	Yes	72 (80.9%)	5 (71.4%)	0.54
	No	17 (19.1%)	2 (28.6%)	

## Data Availability

The data that support the findings of this study are available from the corresponding authors upon reasonable request.
